# A Comprehensive X-ray Dataset for Pediatric Ulna and Radius Fractures Analysis

**DOI:** 10.1038/s41597-026-06666-w

**Published:** 2026-01-28

**Authors:** Suigu Tang, Lihong Ou, Weiheng Li, Zhu Xiong, Ning Li, Huazhu Liu, Yanyan Liang, Zhenhui Zhao

**Affiliations:** 1 School of Integrated Circuits (International School of Microelectronics), Dongguan University of Technology, Dongguan, 523808 China; 2 School of Computer Science and Engineering, Faculty of Innovation Engineering, Macau University of Science and Technology, 999078 Macau, China; 3 Department of Pediatric Orthopedics, Shenzhen Pediatrics Institute of Shantou University Medical College, Shenzhen, 518034 Guangdong China

**Keywords:** Data processing, Radiography

## Abstract

Pediatric forearm fractures, particularly involving the ulna and radius, are among the most common childhood injuries. However, the lack of standardized and openly available datasets has limited progress in artificial intelligence research and constrained clinical validation. To address this issue, we present the Pediatric Ulna and Radius Fractures (PediURF) dataset, a first-of-its-kind, publicly available collection of over 10,000 de-identified images. Each image is carefully annotated by expert radiologists and categorized into three clinically relevant types: proximal, midshaft, and distal fractures. By releasing PediURF, we aim to provide an accessible resource for deep learning-based models development, benchmarking, and clinical training. To validate its utility, we proposed URFNet, a dual-view classification model designed to integrate anteroposterior and lateral perspectives. The proposed model achieved the best performance when compared with other classification models. Collectively, the proposed PediURF dataset provides a valuable foundation for future deep learning-based studies in pediatric fracture classification.

## Background & Summary

Fractures are among the most frequent unintentional injuries in children, with epidemiological studies indicating that nearly half of all children will experience at least one fracture before adulthood^1^. Upper limb fractures constitute approximately 80% of pediatric cases^2,3^, with ulna and radius fractures representing nearly half of all upper limb fracture cases^4^. Diagnostic accuracy, however, is often complicated by the unique characteristics of the immature skeleton, where radiographic appearances vary with bone maturation and fracture patterns. Non-specialist physicians, who are commonly the first to interpret radiographs in emergency settings, may overlook up to 11% of acute pediatric fractures compared with pediatric radiologists, with nearly 8% of these errors leading to changes in patient management^5^. These limitations underscore the need for standardized epidemiological data and openly accessible resources to support clinical care, prevention strategies, and artificial intelligence–based research.

Recent years have witnessed progress in artificial intelligence (AI) for X-ray imaging, with deep learning models showing promising accuracy in pediatric wrist fracture detection. Such models could be useful as an interpretative adjunct where specialist opinions are not always available. For example, Till *et al*. validated the performance of YOLOv7, demonstrating its utility for pediatric wrist fracture detection^6^. Similarly, Ju *et al*. reported encouraging results using the more recent YOLOv8 model^7^. Zech *et al*. further investigated object detection approaches, showing that Faster R-CNN achieved accuracy comparable to that of resident physicians^8^. Building on these advances, Ferdi proposed a Lightweight G-YOLOv11 model designed to reduce computational demands while addressing the biological and temporal complexities unique to pediatric fractures, thereby improving the feasibility of intelligent diagnostic systems^9^.

Beyond wrist-specific tasks, additional studies have explored broader applications of machine learning for pediatric fracture recognition. Zech *et al*. demonstrated that R-CNN and EfficientNetV2-Small achieved an accuracy of 89.7% and an AUC of 0.96^10^. Das *et al*. applied ResNet50 to a dataset of 3,000 pediatric bone X-ray images, reporting an accuracy of 96.5%^11^. More recently, Alam *et al*. combined MobileNet with a tree-based lightweight gradient boosting framework, yielding even higher accuracy for pediatric fracture identification^12^. Collectively, these studies highlight the growing potential of artificial intelligence in this field, while also underscoring the need for standardized, openly available datasets to support reproducibility, benchmarking, and clinical translation.

Although deep learning-based models for X-ray images are increasingly being explored (as shown in Table 1), pediatric fracture datasets—particularly involving the ulna and radius—remain difficult to collect at scale. The difficulties arise from stringent legal and ethical restrictions, as well as variability in clinical data collection and hospital record-keeping practices across institutions. Taken together, the high misinterpretation rate, the variability introduced by skeletal maturation, and the lack of standardized open datasets have collectively limited the reproducibility of AI research and hindered its translation into clinical practice.

**Table 1:** An overview of existing X-ray datasets.

Dataset	Year	Size	Location	Task	Availability
VinDr-SpineXR^23^	2021	10,466	Spinal bone	Classification Detection	Public
PelviXNet^24^	2021	5,204	Pelvic	Detection	Private
GRAZPEDWRI-DX^25^	2022	20,327	Wrist	Detection	Public
PA Pediatric Wrist^26^	2022	2,474	Distal radius	Classification	Private
FracAtlas^27^	2023	4,083	Bone fracture	Detection	Public
PediCXR^28^	2023	9,125	Chest	Detection	Public
X-ray Bone Fracture^29^	2024	16,061	Bone fracture	Classification Segmentation	Public
CSXA^30^	2024	4,963	Cervical spine	Classification	Public
Panoramic Mandibular Fracture images^31^	2024	498	Mandible	Classification Detection	Private
BIDMC Proximal Femur Radiograph^32^	2024	823	Proximal femur	Classification Detection	Private

To address these problems, we introduce the Pediatric Ulna and Radius Fractures (PediURF) dataset, a comprehensive X-ray image dataset specifically curated for pediatric ulna and radius fractures. PediURF contains high-quality X-ray images with detailed annotations by experienced radiologists. The dataset is designed to serve multiple purposes: it provides a standardized benchmark for developing and validating deep learning models, supports reproducibility in computational research, and offers a valuable resource for epidemiological studies and clinical education.

By openly releasing PediURF, we aim to lower the barriers to entry for researchers, enable fair comparison of methods, and promote collaboration across the pediatric radiology and machine learning communities. In addition, we provide some classification models trained on PediURF to serve as reference points for future studies, allowing researchers to build upon established performance benchmarks. We anticipate that PediURF will not only complement existing datasets but also advance the field by enabling more robust, clinically relevant, and generalizable AI solutions for pediatric fracture care.

## Methods

**Fig. 1:**
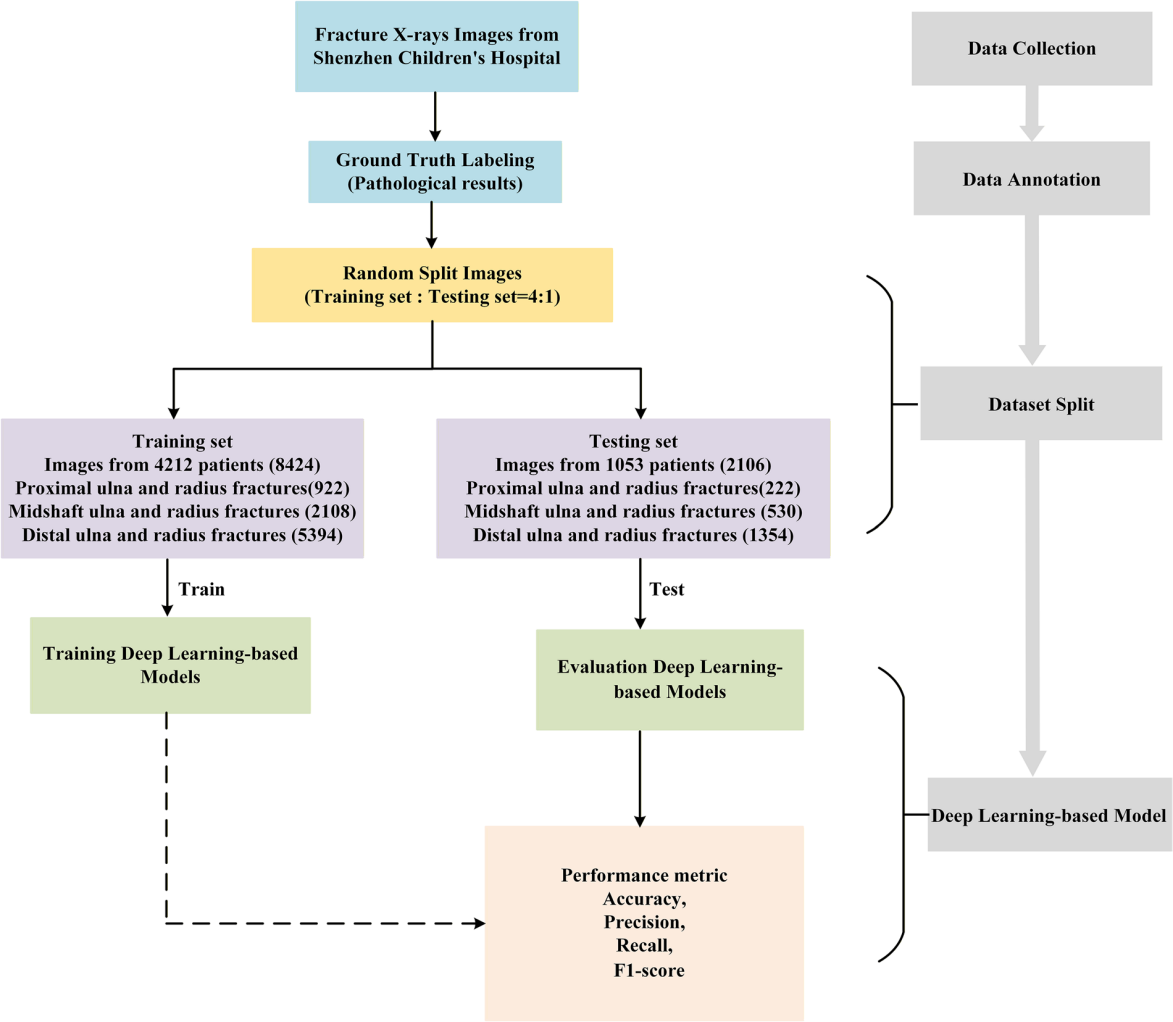
The workflow of the PediURF dataset.

The overall construction workflow of the PediURF dataset is presented in Fig. 1. The PediURF dataset was first constructed from pediatric forearm fracture examinations collected at Shenzhen Children’s Hospital between 2013 and 2024. Each case includes anteroposterior and lateral X-ray images, and is stored in o JPEG (.jpg) format. Using the JPEG format can significantly reduce file size and help speed up download, storage, and access, thus benefiting a wider range of users and computing environments.

While the JPEG format is a convenient and widely used image format, its lossy compression can lead to the loss of fine structural information crucial for clinical assessment and algorithmic analysis. Furthermore, JPEG files lack metadata commonly found in formats like DICOM, such as acquisition parameters and demographic information. To help mitigate these limitations, we provide the supplementary files (train.csv and test.csv) containing key non-identifiable metadata, including patient age and gender.

All X-ray images underwent strict de-identification to protect patient privacy. Personal identifiers, including patient ID, name, and date of birth, were completely removed before dataset release. Ethical approval for the study was obtained from the Ethics Committee of Shenzhen Children’s Hospital (Approval No.: 202505902), where consent for participation and data sharing was obtained from a parent or guardian (for children). All procedures involving human participants were conducted in accordance with the Declaration of Helsinki. The approval allows the collection and analysis of the data and permits the open publication of the fully de-identified dataset for research and educational use.

After collecting the dataset, the workflow proceeds through three key stages: data annotation, dataset split, and deep learning-based models.

### Data annotation

**Fig. 2:**
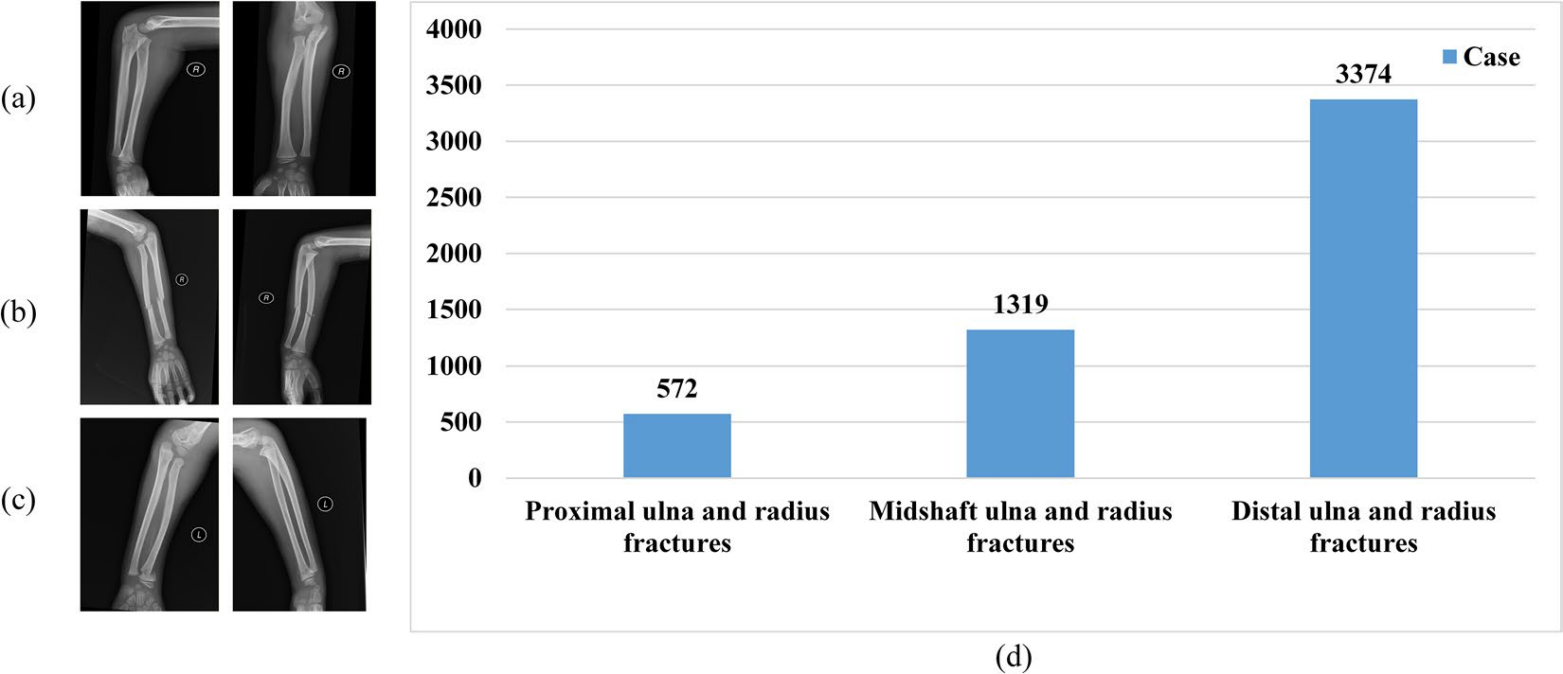
Types and numbers of ulna and radius fractures in the dataset. (**a**) Proximal ulna and radius fractures. (**b**) Midshaft ulna and radius fractures. (**c**) Distal ulna and radius fractures. (**d**) Number of ulna and radius fractures.

Annotations were performed by experienced radiologists and validated using clinical reports and pathological findings to ensure accurate labeling of fracture types. This ensures that the classification of fracture types is clinically accurate and reliable, providing a strong foundation for subsequent deep learning experiments. Cases were categorized into three clinically relevant categories: proximal, midshaft, and distal ulna and radius fractures. Anatomically, the segment nearest to the elbow joint is defined as proximal, as it lies closest to the heart, whereas the segment near the wrist is defined as distal, being the farthest from the heart. The midshaft category corresponds to fractures located in the central portion of the forearm. Therefore, it can be observed from Fig. 2 that the types and numbers of ulna and radius fractures are in the proposed dataset.

All radius and ulna fracture types have both anteroposterior (front.jpg) and lateral (side.jpg) images, providing comprehensive visual perspectives necessary for accurate diagnosis and classification (see in Fig. 2(a–c)). Radiography is the primary imaging modality for detecting ulna and radius fractures in children, and both anteroposterior and lateral views are typically required. These two projections provide complementary information to aid in the accurate localization and characterization of the fracture. In some cases, a fracture may not be apparent on one view but clearly visible on another, particularly when angular displacement is present. Because fractures in this region are often accompanied by three-dimensional variations (including displacement, angulation, and rotation), evaluation with a single projection is insufficient. While the anteroposterior view is primarily used to assess lateral displacement, fracture line morphology, and changes in the radioulnar space, the lateral view is crucial for identifying anteroposterior displacement, angulation, and rotational deformity. The combined use of these views allows for a comprehensive assessment of the fracture characteristics and provides a reliable basis for guiding reduction and fixation strategies.

Specifically, Fig. 2(a) illustrates proximal ulna and radius fractures, which are located near the elbow joint. These fractures are less frequently observed but are clinically challenging due to their proximity to growth plates and joint structures. Figure 2(b) shows midshaft ulna and radius fractures, typically situated in the central portion of the forearm where mechanical stresses and traumatic impacts are most often concentrated. Figure 2(c) presents distal ulna and radius fractures, which are the most common type and are usually found near the wrist joint.

Additionally, the dataset distribution is shown in Fig. 2(d). The statistics indicate a clear imbalance among categories: proximal ulna and radius fractures are relatively rare, with only 572 cases, whereas distal ulna and radius fractures represent the majority, with 3,374 cases. Midshaft ulna and radius fractures fall in between, with a total of 1,319 cases. This distribution reflects the clinical prevalence of fracture sites in pediatric populations, as distal fractures are most frequently observed in practice.

Taken together, these results reveal both the diversity of fracture types in the PediURF dataset and their inherent clinical distribution patterns. This dual perspective, combining visual examples with statistical overviews, makes the dataset a valuable benchmark for developing robust deep learning models for classifying ulna and radius fractures in children.

### Dataset split

Following expert annotation, the proposed PediURF dataset was randomly divided into training and testing subsets at a ratio of 4:1. To ensure patient-level independence and avoid data leakage, all X-ray images belonging to the same patient were assigned exclusively to a single subset. Each case includes both front and side images, resulting in a training set comprising 4,212 pediatric patients with a total of 8,424 images. Specifically, the training set contained 922 proximal ulna and radius fractures, 2,108 midshaft ulna and radius fractures, and 5,394 distal ulna and radius fractures. The testing set consisted of 1,053 patients with 2,106 images, including 222 proximal, 530 midshaft, and 1,354 distal fractures. Stratified sampling was applied to preserve the clinical distribution of fracture categories across subsets. This distribution reflects real-world prevalence in pediatric populations, where distal fractures are substantially more common than proximal or midshaft fractures.

The training subset is used to optimize deep learning-based models, which learns discriminative representations of the fracture images. Once trained, the model is evaluated on the independent testing set to assess its ability to generalize to unseen patient data. The performance assessment relies on widely used evaluation metrics in medical image analysis, including Accuracy, Precision, Recall, and F1-score. These metrics provide a comprehensive understanding of the classification ability of the model, balancing overall correctness with sensitivity and robustness across different fracture categories. As a result, this experimental pipeline establishes a rigorous and clinically meaningful framework for developing.

### Deep learning-based model

To demonstrate how the PediURF dataset can be used in downstream tasks, we provide a simple example based on a deep learning classification workflow. In this example, each case is represented by paired anteroposterior and lateral X-ray images, which are provided as inputs to the models. The two images are processed in parallel with two feature extractors, and the resulting features are combined to generate a single prediction of the fracture. This setup shows how the paired-view structure of the dataset can be exploited in downstream studies.

**Fig. 3:**
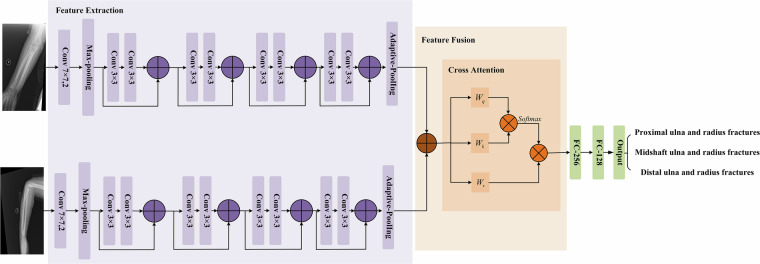
The overall architecture of URFNet.

In this illustrative example, the Ulna and Radius Fractures Network (URFNet) was developed for pediatric fracture classification (Fig. 3) and is designed to jointly process anteroposterior and lateral X-ray images to capture complementary information from both views. Each view is passed through an identical convolutional stream consisting of an initial 7 × 7 convolution and max-pooling layer, followed by multiple 3 × 3 residual blocks with skip connections to progressively extract higher-level semantic features while preserving anatomical detail. Adaptive pooling is applied to standardize feature map sizes across inputs.

The feature maps extracted from the two parallel streams are then fused using a cross-attention mechanism. In this step, Query (Q), Key (K), and Value (V) embeddings are derived from the combined features through learnable projections, and scaled dot-product attention enables each view to attend to complementary cues from the other. The fused features are subsequently transformed by fully connected layers and used to classify fractures into three anatomical categories: proximal ulna and radius fractures, midshaft ulna and radius fractures, and distal ulna and radius fractures.

## Data Records

**Fig. 4:**
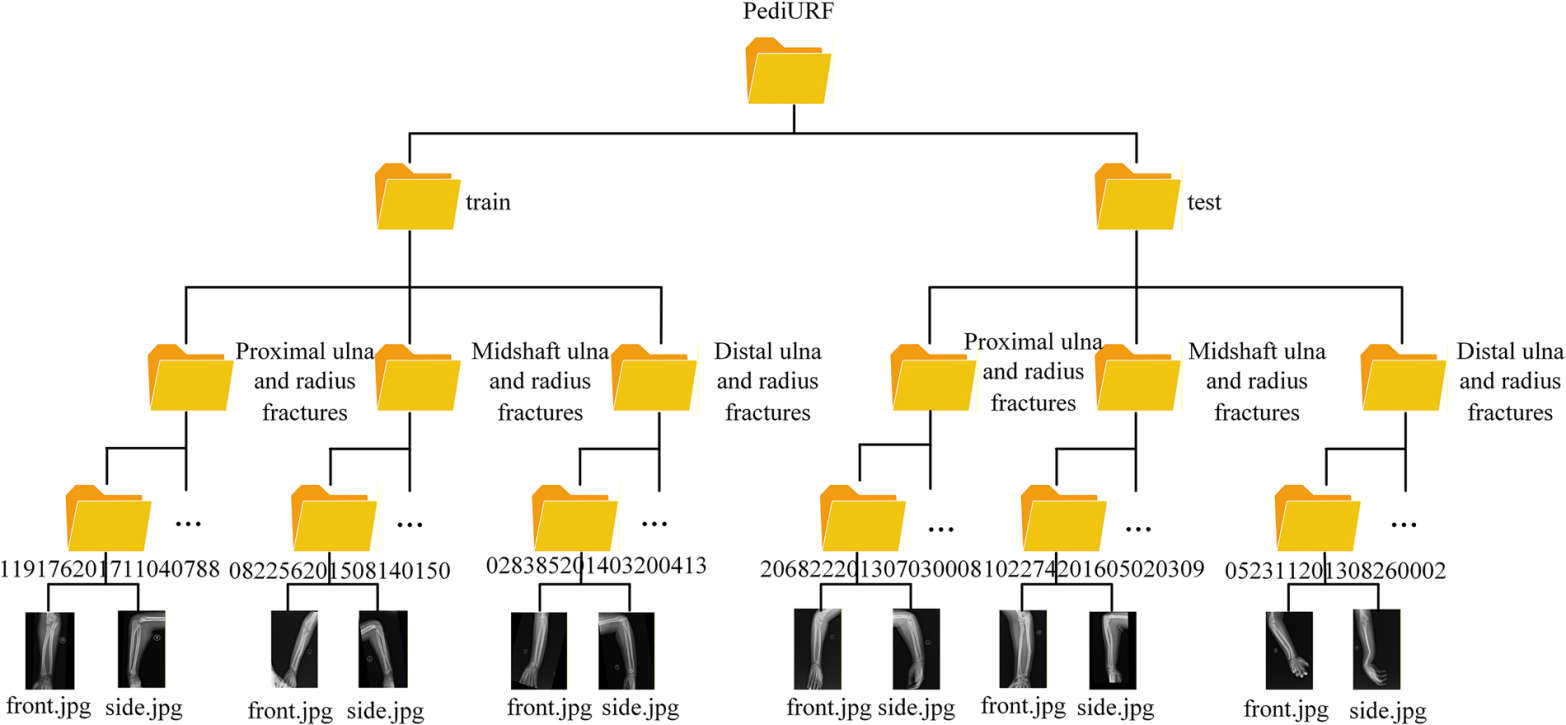
The folder structure of the PediURF dataset.

The dataset generated and analyzed in this study is publicly available on Figshare and is publicly available at the following [10.6084/m9.figshare.29998954]^13^. The hierarchical organization of the PediURF dataset is structured for pediatric ulna and radius fracture classification tasks, as shown in Fig. 4. At the root level, the dataset is divided into two main subsets: train and test, ensuring a standardized split for model training and evaluation. Within each subset, the data is further categorized into three anatomical fracture types: Proximal ulna and radius fractures, Midshaft ulna and radius fractures, and Distal ulna and radius fractures. This classification reflects the clinical distinctions in fracture location along the forearm bones, which are critical for accurate diagnosis and treatment planning.

Inside each category, individual patient cases are stored in separate folders labeled with unique identifiers, such as “119176201711040788” or “082256201508140150.” Each folder contains a pair of X-ray images, typically named front.jpg and side.jpg, representing different imaging perspectives (anteroposterior and lateral). This dual-view design provides a more comprehensive view of the fracture, allowing the model to learn richer features about the fracture.

Overall, this dataset organization supports robust supervised learning by clearly separating data into training and testing partitions, offering balanced anatomical coverage, and incorporating multiple imaging angles per case. Such a structured design not only facilitates reproducibility in experimental setups but also enhances the clinical relevance of deep learning-based models trained on PediURF.

## Technical Validation

### Dataset quality and consistency

A series of quality assurance steps was applied to ensure the consistency and technical reliability of the PediURF dataset. Fracture labels were reviewed with reference to anatomical landmarks, and cases with uncertain fracture localization were re-examined until agreement was reached, thereby reducing inter-observer variability.

Consistency checks were further conducted at the case level. Each record was confirmed to contain a complete and correctly paired set of anteroposterior and lateral X-ray images, and incomplete or mismatched entries were removed. To prevent information leakage across subsets, patient-level identifiers were used to ensure that all images from the same individual were assigned to a single data split. Together, these procedures support the internal coherence and reproducibility of the dataset.

### Illustrative deep learning examples

As a complementary illustration of downstream dataset reuse, we conducted a series of representative deep learning experiments to demonstrate that the PediURF dataset can support a range of commonly used classification architectures.

To optimize model performance, we used the cross-entropy loss function and the SGD optimizer with a learning rate of 0.001, a batch size of 32, and a training epoch of 200. Additionally, we used a StepLR learning rate scheduler, which smoothly decays the learning rate to 0.0005 over the course of 100 training epochs, aiming to improve the stability and convergence speed of the training process. The pre-trained models on ImageNet are used, and our proposed model uses the ResNet34 pre-trained model. All images were standardized to a resolution of 224 × 224. To reduce the risk of overfitting during all model training, data augmentation techniques, including random horizontal and vertical flips, each with a probability of 0.5. Additionally, with a probability of 0.8, appearance-based augmentations were introduced, comprising adjustments to brightness, contrast, and saturation (each sampled from the range 0.8–1.2), as well as a hue shift sampled from −0.05 to 0.05. For dual-view inputs, all augmentation parameters were applied synchronously to both images to preserve anatomical consistency across views. Finally, all experiments were conducted using an NVIDIA GeForce RTX 4090 Ti GPU.

**Fig. 5:**
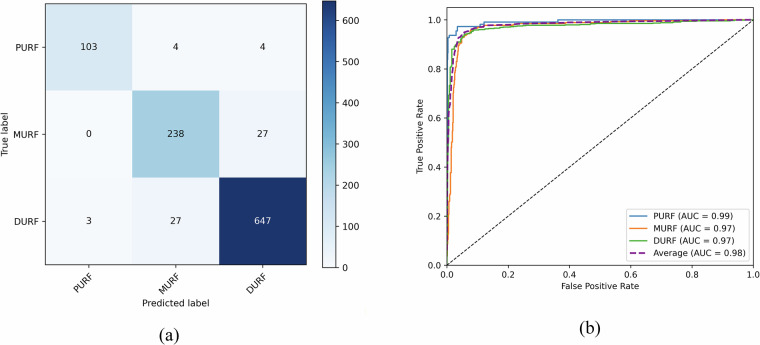
(**a**) Confusion matrix. (**b**) ROC curves. PURF: Proximal ulna and radius fractures. MURF: Midshaft ulna and radius fractures. DURF: Distal ulna and radius fractures.

The confusion matrix and ROC curves are presented in Fig. 5. As shown in the figure, most fractures were correctly identified by the proposed model. All three categories demonstrated strong discriminative performance. These consistently high AUC values indicate that the model remains robust across all classes despite the inherent imbalance in the dataset.

Besides, PediURF was used to train several conventional convolutional neural networks (CNNs) and transformer-based models, including AlexNet^14^, VGG16^15^, VGG19^15^, ResNet18^16^, ResNet34^16^, ResNet50^16^, DenseNet121^17^, DenseNet169^17^, MobileNet_V2^18^, MobileNet_V3_Small^18^, EfficientNet_B0^19^, EfficientNet_B1^19^, ViT-Ti^20^, ViT-S^20^, ViT-B^20^, ViT-L^20^, DeiT-Ti^21^, DeiT-S^21^, DeiT-B^21^, EfficientFormer-L1^22^, and EfficientFormer-L3^22^. Since the contrast model can only input a single image, we mix the anteroposterior and lateral X-ray images as the model input.

A comprehensive comparison of multiple deep learning models on a fracture classification task, reporting four key evaluation metrics: accuracy, precision, recall, and F1-score, is shown in Table 2. It can be seen from Table 2 that our proposed URFNet achieved the best overall performance, significantly outperforming CNN- and Transformer-based baseline models.

**Table 2:** All model classification results.

Models	Accuracy(%)	Precision(%)	Recall(%)	F1-score(%)
AlexNet^14^	73.43 ± 0.29	75.20 ± 1.64	51.93 ± 0.42	61.43 ± 0.59
VGG16^15^	83.91 ± 1.24	81.36 ± 1.66	71.97 ± 2.89	76.37 ± 2.25
VGG19^15^	85.09 ± 0.47	82.29 ± 1.17	74.09 ± 2.24	77.95 ± 1.13
ResNet18^16^	80.27 ± 0.94	82.12 ± 0.79	62.82 ± 1.96	71.18 ± 1.37
ResNet34^16^	85.01 ± 1.15	84.14 ± 1.27	74.75 ± 2.18	79.16 ± 1.74
ResNet50^16^	67.13 ± 1.16	47.09 ± 0.50	37.86 ± 1.92	41.95 ± 1.27
DenseNet121^17^	86.64 ± 0.64	85.90 ± 0.92	77.28 ± 1.87	81.35 ± 1.21
DenseNet169^17^	64.61 ± 0.29	37.92 ± 2.79	34.15 ± 0.58	35.91 ± 1.56
MobileNet_V2^18^	66.61 ± 0.50	46.58 ± 0.76	37.00 ± 0.70	41.24 ± 0.67
MobileNet_V3_Small^18^	69.06 ± 1.07	79.10 ± 1.18	42.01 ± 1.65	54.87 ± 1.65
EfficientNet_B0^19^	79.01 ± 1.64	73.33 ± 2.73	69.68 ± 2.24	71.45 ± 2.42
EfficientNet_B1^19^	65.65 ± 1.13	65.35 ± 4.30	38.01 ± 2.56	47.98 ± 2.19
Vit_Ti^20^	85.77 ± 0.83	83.65 ± 0.98	78.90 ± 1.64	81.20 ± 1.27
Vit_S^20^	88.22 ± 0.45	87.15 ± 0.34	83.08 ± 1.30	85.06 ± 0.74
Vit_B^20^	88.14 ± 1.54	86.20 ± 1.32	83.06 ± 3.34	84.60 ± 2.37
Vit_L^20^	89.25 ± 1.34	87.30 ± 1.11	85.24 ± 2.85	86.25 ± 1.90
Deit_Ti^21^	83.58 ± 1.92	81.41 ± 2.05	72.86 ± 4.33	76.88 ± 3.33
Deit_S^21^	84.61 ± 2.10	83.02 ± 3.50	75.49 ± 3.56	79.07 ± 3.51
Deit_B^21^	85.53 ± 0.73	85.90 ± 0.77	74.39 ± 1.19	79.73 ± 0.91
EfficientFormer_L1^22^	66.74 ± 0.97	76.50 ± 1.44	38.25 ± 1.65	51.00 ± 1.76
EfficientFormer_L3^22^	66.99 ± 0.62	44.22 ± 0.87	38.27 ± 1.02	41.03 ± 0.90
URFNet	**93.51** ± **0.33**	**92.46** ± **0.81**	**92.82** ± **0.40**	**92.63** ± **0.47**

All results were obtained by training each model five times with different random seeds and reporting the mean ± standard deviation to ensure statistical stability.

Following the illustrative classification example, we briefly explored the role of feature fusion by contrasting a cross-attention mechanism with simple concatenation (Table 3). This comparison is included solely to demonstrate how different fusion strategies may be applied when using dual-view inputs in the PediURF dataset.

**Table 3:** Ablation study.

Feature fusion	Accuracy(%)	Precision (%)	Recall(%)	F1-score(%)
Concatenation	93.22 ± 0.37	92.42 ± 0.55	92.08 ± 0.59	92.25 ± 0.53
Cross-attention	**93.51** ± **0.33**	**92.46** ± **0.81**	**92.82** ± **0.40**	**92.63** ± **0.47**

All results were obtained by training each model five times with different random seeds and reporting the mean ± standard deviation to ensure statistical stability.

Finally, as part of the illustrative example, we report several characteristics to provide a general indication of the computational scale associated with the model configuration used. In this setup, the URFNet comprises approximately 21.45 M parameters and requires about 7.36GFLOPs per forward pass. Under the experimental setup described above, the average training time per epoch was roughly 66 s, and inference for a single case was completed within a few milliseconds. These values are reported for reference only and serve to illustrate the practical feasibility of applying the dataset within common deep learning workflows in real-world clinical environments.

The network design and implementation details are included solely to illustrate how the dataset can be used in practice; within this illustrative setting, the dual-view (anteroposterior and lateral) design demonstrates superior performance over single-image (anteroposterior or lateral) input by effectively exploiting complementary features from multiple views.

In summary, the proposed PediURF dataset is the first publicly available resource specifically designed for the classification of pediatric forearm fractures. An advantage of PediURF is that each case includes both anteroposterior and lateral X-ray images. By integrating these two views, PediURF more accurately reflects real-world diagnostic practice and supports the development of deep learning-based models that can robustly handle multi-view clinical data.

Pediatric X-ray images present several inherent challenges that can affect both manual interpretation and automated analysis. Variations in projection angles are common in clinical settings, particularly among children who may have difficulty maintaining stable positioning. Differences in limb rotation, flexion, or centering can alter the visibility of fracture lines and introduce variability that complicates model training. Additionally, the appearance of the epiphyseal plate changes markedly with age, and subtle differences in ossification patterns may either mimic or obscure true fracture features, further increasing diagnostic complexity. Heterogeneity in acquisition protocols and imaging equipment across institutions can also influence image contrast, sharpness, and overall quality.

Despite these limitations, the clinical potential of automated fracture detection systems remains substantial. Models such as URFNet could serve as valuable decision-support tools by assisting general practitioners or emergency physicians with rapid triage, highlighting suspicious regions on radiographs, or providing a second opinion in cases where fractures are subtle or easily missed. Such approaches may be especially beneficial in resource-limited settings or high-volume clinical environments where timely and accurate interpretation is essential. Looking ahead, incorporating multi-center validation and additional annotation types—such as fracture localization—will further enhance the robustness and generalizability of these methods.

## Usage Notes

The PediURF dataset is distributed in a simple and accessible format to facilitate its use by researchers. All X-ray images are provided as JPEG files and can be obtained directly from the public repository. Once downloaded, the images may be opened with standard libraries such as PIL or OpenCV. Because each case includes both anteroposterior and lateral projections, users should take care to correctly pair the two views when incorporating the data into model development workflows.

## Data Availability

The proposed dataset collected in this work has been fully de-identified and released under an open-access license to support reproducibility and reuse. The dataset is available at 10.6084/m9.figshare.29998954.

## Code availability

The source code for the proposed URFNet model, together with the complete experimental framework, is openly accessible at https://github.com/GG-Tang/URFNet.

The repository includes all scripts necessary to replicate the results of this study. The main directory provides implementations for the proposed URFNet construction (models.py), dataset handling and preprocessing (datajson.py and dataset.py), as well as training (train.py) and evaluation (test.py) procedures. In addition, a dedicated folder named Comparison models contains reference implementations of baseline models, enabling direct benchmarking of URFNet against widely used convolutional and transformer-based networks.

To facilitate reproducibility, the repository also provides a requirements.txt file that lists the required Python dependencies, ensuring consistency across different computing environments. By making this implementation freely available, we aim to ensure transparency, allow independent verification of our findings, and promote further development of deep learning methods for pediatric fracture classification.
